# Using the R = MC^2^ heuristic to understand barriers to and facilitators of implementing school-based physical activity opportunities: a qualitative study

**DOI:** 10.1186/s12889-024-17744-2

**Published:** 2024-01-17

**Authors:** Derek W. Craig, Timothy J. Walker, Paula Cuccaro, Shreela V. Sharma, Natalia I. Heredia, Michael C. Robertson, Maria E. Fernandez

**Affiliations:** 1 Department of Health Promotion & Behavioral Sciences, Center for Health Promotion and Prevention Research, The University of Texas Health Science Center at Houston School of Public Health, Houston, TX USA; 2 Department of Epidemiology, Human Genetics, & Environmental Sciences, Center for Health Equity, Michael & Susan Dell Center for Healthy Living, The University of Texas Health Science Center at Houston School of Public Health, Houston, TX USA; 3 Department of Nutrition, Metabolism, and Rehabilitation Sciences, The University of Texas Medical Branch at Galveston, Galveston, TX USA; 4 Department of Family and Preventive Medicine, TSET Health Promotion Research Center, University of Oklahoma Health Sciences Center, Oklahoma City, OK USA

**Keywords:** Physical activity, Implementation, Organizational readiness, Schools

## Abstract

**Background:**

Schools are a key setting for supporting youth physical activity, given their broad reach and diverse student populations. Organizational readiness is a precursor to the successful implementation of school-based physical activity opportunities. The R = MC^2^ heuristic (Readiness = Motivation x Innovation-Specific Capacity x General Capacity) describes readiness as a function of an organization’s motivation and capacity to implement an innovation and can be applied to better understand the implementation process. The purpose of this study was to explore the barriers to and facilitators of implementing school-based physical activity opportunities in the context of organizational readiness.

**Methods:**

We analyzed interview data from 15 elementary school staff (principals, assistant principals, physical education teachers, and classroom teachers) from a school district in Texas. We focused on factors related to adopting, implementing, and sustaining a variety of school-based physical activity opportunities. We used the Framework Method to guide the analysis and coded data using deductive (informed by the R = MC^2^ heuristic) and inductive approaches. Themes were generated using the frequency, depth, and richness of participant responses.

**Results:**

Four themes emerged from the data: (1) implementation is aided by the presence of internal and external relationships; (2) physical activity opportunities compete with other school priorities; (3) seeing the benefits of physical activity opportunities motivates school staff toward implementation; and (4) staff buy-in is critical to the implementation process. Themes 1–3 aligned with subcomponents of the R = MC^2^ heuristic (intra- and inter-organizational relationships, priority, and observability), whereas Theme 4 (staff buy-in) related to multiple subcomponents within the Motivation component but was ultimately viewed as a distinct construct.

**Conclusion:**

Our results highlight and explain how key readiness constructs impact the implementation of school-based physical activity opportunities. They also highlight the importance of obtaining staff buy-in when implementing in the school setting. This information is critical to developing readiness-building strategies that help schools improve their capacity to deliver physical activity opportunities effectively.

**Trial registration:**

Not applicable.

**Supplementary Information:**

The online version contains supplementary material available at 10.1186/s12889-024-17744-2.

## Background

The education sector plays a key role in supporting youth physical activity. Schools (K–12) serve a diverse student population and have a broad reach, with approximately 48.1 million students enrolled in public schools in the United States [1]. In addition, students spend a significant amount of time each day (6–8 h) at school, which underscores the importance of providing high-quality physical activity opportunities. The Institute of Medicine and the World Health Organization recommend schools use a whole-of-school approach for physical activity promotion [2, 3]. This multi-component approach includes offering physical activity opportunities before, during, and after school through means such as physical education (PE), recess, classroom-based activities, active transportation to/from school, and intra/extramural sports. Providing a variety of opportunities throughout the school day can create an environment that promotes physical activity and helps students attain the nationally recommended 60 min of daily physical activity [4–7]. However, coordinating the implementation of multiple physical activity opportunities can be challenging for many schools.

There are many factors that influence successful implementation in the school setting. Research has found that district/school support [8–10], clear communication [11–14], staff characteristics (e.g., motivation, buy-in) [15–17], and community partnerships [9, 14, 17, 18] have a positive impact on the implementation of school-based physical activity opportunities. Conversely, competing priorities [11, 19–21], lack of resources [8, 9, 21], and staff capacity for implementation [22–24], have frequently been cited as implementation barriers across all school types (i.e., elementary, middle, and high). Understanding implementation barriers and facilitators can help schools address the factors that have an impact on their ability and capacity to successfully adopt, implement, and sustain physical activity opportunities [25].

Organizational readiness is a multi-faceted construct found in several prominent implementation science theories and frameworks [26–30]. Scaccia et al. conceptualized readiness using the R = MC^2^ heuristic (Readiness = Motivation x Innovation-Specific Capacity x General Capacity), which describes readiness as the combination of an organization’s motivation and capacity to implement an innovation (e.g., intervention or practice change) [27]. The R = MC^2^ heuristic is a determinants framework [31] that can be used to identify organizational-level barriers and facilitators that influence implementation outcomes. This is an essential first step to understanding the role organizational readiness plays throughout each phase of implementation (i.e., pre-implementation, adoption, active implementation, and sustainability). Nonetheless, the R = MC^2^ heuristic has seldom been used to qualitatively explore organizational readiness [32–34]. Studies applying the R = MC^2^ heuristic, particularly those examining school-based physical activity implementation, will provide much-needed information on the relevancy of readiness constructs and inform which constructs to prioritize when developing strategies to build organizational readiness. Therefore, the purpose of this study was to identify barriers to and facilitators of implementing school-based physical activity opportunities within the context of organizational readiness as defined by the R = MC^2^ heuristic.

## Methods

### Study design and setting

We conducted a secondary analysis of qualitative data collected in the spring of 2018. Recruitment was conducted among elementary school staff from a culturally diverse, urban school district in southeast Texas. Overall, the district educates approximately 35,000 students annually. The district’s elementary school student population was predominantly Hispanic (58.0%), and nearly two-thirds (61.6%) were considered economically disadvantaged at the time of the study [35]. The Committee for the Protection of Human Subjects (CPHS) at the University of Texas Health Science Center at Houston (UTHealth Houston) and the school district’s Research and Evaluation office approved this study.

### Recruitment & data collection (parent study)

The parent study conducted a series of semi-structured individual interviews which created a qualitative dataset from which multiple papers have been published [12, 36–38]. The primary purpose of these interviews was to obtain a greater understanding of physical activity practices within the district’s elementary schools. The parent study research team worked with the district’s wellness team to develop and coordinate a recruitment plan that employed a purposeful sampling approach to identify and select school staff to participate in interviews [39]. The district wellness staff contacted elementary school staff (principals, assistant principals, PE teachers, and classroom teachers) about the parent study, confirmed they possessed knowledge of the physical activity programming that took place within their respective schools, and then provided the parent study research team with a list of contact information for school staff interested in participating. The research team followed up with interested staff members to schedule an in-person interview. After completing each interview, participants were asked to recommend two to three colleagues to contact for recruitment. Recruitment was balanced to enroll approximately equal numbers of participants across the four job types.

Interviews were selected as the optimal method for capturing individual perspectives on the implementation of a range of physical activity opportunities across multiple job types in addition to addressing logistical challenges with scheduling during school hours. Interview questions explored staff perspectives of physical activity in schools and included specific probes about implementation barriers and facilitators (see Supplemental File). Questions and probes were approved by the district wellness team and refined during the initial interviews for clarity and relevance. A total of 22 school staff were invited to participate in the study. Interviews were completed with 15 elementary school staff (principals, *n* = 4; assistant principals, *n* = 3; PE teachers, *n* = 4; and classroom teachers, *n* = 4); 2 individuals declined to participate, and the remaining 5 individuals did not respond to multiple contact attempts. The sample was predominantly female (93%), and participants had been working in their current position for an average of 8.5 years. Participants represented 40% of the elementary schools in the district (*n* = 10), nine of which were considered Title 1 (i.e., a minimum of 40% of the students served by the school are eligible for a free or reduced-price lunch). Interviews lasted approximately 45 min and were audio recorded and professionally transcribed verbatim. Written informed consent was obtained from participants before the interviews were conducted, and each participant received a $30 gift card.

### Data analysis (current study)

The current study is a secondary analysis of the qualitative data collected in the parent study. We used the Framework Method [40, 41] to identify key issues and generate themes to better understand the barriers to and facilitators of implementing school-based physical activity opportunities. Three members of the research team coded the transcripts, including using a general code to identify any conversation related to implementation, which we defined as “the process of putting physical activity opportunities to use within the school setting” [42]. Then, the lead author used deductive and inductive approaches to code the implementation-specific excerpts. For the deductive coding, we used a predefined list of subcomponents from the R = MC^2^ heuristic (Table 1). The inductive coding allowed for the identification of additional concepts not contained within the R = MC^2^ heuristic. The coders met regularly to discuss the application of deductive and inductive codes and develop a qualitative codebook to enhance data dependability. After charting the data, we generated themes based on the frequency, depth, and richness of participants’ responses. Then, we assessed the overlap between the emergent themes and the R = MC^2^ subcomponents (deductive codes) to identify relevant concepts not currently contained within the R = MC^2^ heuristic. The coders invited feedback from senior co-authors with qualitative expertise (TW, PC, MEF) during the theme generation and comparison processes to ensure confirmability of the qualitative data. Data were coded, organized, and interpreted using Microsoft Excel and ATLAS.ti (Version 9) [43].

**Table 1:** R = MC^2^ Heuristic: Components, Subcomponents, and Definitions

Component	Subcomponent	Definition
General Capacity	Organizational innovativeness	Openness to change in general.
	Resource utilization	Ability to acquire and allocate resources, including time, money, effort, and technology.
	Organizational culture	Norms and values of how things are done in our organization.
	Organizational climate	The feeling of being part of this organization.
	Leadership	Effectiveness of the organization’s leaders.
	Learning climate	The feeling of having enough time for reflection after trying something new.
	Staff capacities	Having enough of the right people to get things done.
	Organizational structure	The method by which work flows through an organization.
Innovation-specific capacity	Knowledge, skills, and abilities	Sufficient abilities to do the innovation.
	Program champion	A well-connected person who supports and models the innovation.
	Implementation climate	The extent to which the innovation will be rewarded, supported, and expected within an organization.
	Inter-organizational relationships	Relationships between organizations that support the innovation.
	Intra-organizational relationships	Relationships within the organization that support the innovation.
Motivation	Simplicity	The innovation seems simple to use.
	Priority	The innovation is important compared to other things the setting does.
	Relative advantage	The innovation seems better than what the setting is currently doing.
	Compatibility	The innovation fits with how the setting does things.
	Trialability	The degree to which the innovation can be tested and experimented with.
	Observability	Ability to see that the innovation is leading to desired outcomes.

## Results

Participants discussed numerous barriers and facilitators to implementing a range of physical activity opportunities, including PE, recess, classroom-based physical activities, active transportation, before- and after-school programs, and singular health-promotion events. Specific to readiness, each subcomponent from the R = MC^2^ heuristic was coded at least twice (range = 2–103). The most frequently coded subcomponents were inter-organizational relationships (*n* = 103), resource utilization (*n* = 99), intra-organizational relationships (*n* = 80), and priority (*n* = 63). In contrast, organizational climate (*n* = 2), relative advantage (*n* = 7), and simplicity (*n* = 9) were coded the least.

Four themes emerged from the deductive and inductive coding: (1) implementation is aided by the presence of internal and external relationships; (2) physical activity opportunities compete with other school priorities; (3) seeing the benefits of physical activity opportunities motivates school staff toward implementation; and (4) staff buy-in is critical to the implementation process. Themes 1–3 (importance of internal and external relationships, competing priorities, and seeing benefits of physical activity opportunities) were comparable to the R = MC^2^ heuristic components of intra- and inter-organizational relationships, priority, and observability, respectively. For Theme 4, staff buy-in (defined as ‘the acceptance of and willingness to support and participate in implementation”) was found to relate to multiple R = MC^2^ subcomponents but was ultimately viewed as a distinct construct.

### Theme 1: Implementation is aided by the presence of internal and external relationships

Relationships among school staff, district employees, and community stakeholders facilitated the implementation of school-based physical activity opportunities. Staff utilized teacher associations, collaborative groups, and departmental trainings to leverage resources, network with colleagues, and share ideas about different physical activity opportunities. A principal stated:

They’re learning the latest of what’s the newest thing coming, whether it’s Playworks, motor labs, anything along those lines. And then they come back [from trainings] and share that with the staff and share that with the kids. That goes on all year.

Campuses rotated hosting in-person meetings, which allowed staff to observe and discuss how various opportunities were being implemented. Connecting with district colleagues was viewed as a highly valuable resource by most participants, as it reduced the amount of time spent researching opportunities and simplified the decision-making process for school leaders. As noted by a classroom teacher:

Yeah, we, the district, about—I want to say three years ago—we started meeting as a collaborative, as a cooperative group from all the campuses, and we met once a month just to talk about the activities we would include in our [motor] labs and how we would change stations and activities and ideas to draw more teachers in.

Relationships with community partners (e.g., non-profit organizations, parent-teacher associations) also supported implementation. The district had long-standing relationships with several local non-profits, which allowed schools to promote a variety of physical activity opportunities to students. Schools developed partnerships with local community centers to address the critical need for afterschool care which had an impact on schools that serve lower-income students and families. These relationships allowed students to use a center’s facilities to be physically active. A PE teacher explained:

But with [a local non-profit organization], we’re just there, and it’s someone else teaching it. Like the YMCA, we will bring the kids there, but they’ll have their certified lifeguards and trainers teaching the kids. [Another local non-profit organization]—actually, with that, we just put them on a bus and send them to [the non-profit organization].

Another PE teacher stated:

We are going to have soccer after school starting next week, once a week, which is a free program that’s not cost-based. They will have karate; they have kickball games. We did have a free program offered from January up until just this month; last week was [the] last day right before spring break. Kids could come in, and [the instructor] would organize different games for the kids, kickball, and it was free. And again, that was through [a non-profit organization].

### Theme 2: Physical activity opportunities compete with other school priorities

Participants discussed numerous priorities with which physical activity opportunities regularly compete. As stated by a classroom teacher, “We just have so many—we’re juggling so many things. It’s hard to know which one has priority.” Standardized test scores were commonly cited as a reason why physical activity opportunities received less attention or were not implemented. For example, A PE teacher stated, “As far as promoting activity, sadly, I don’t think it’s on the top of too many people’s priority lists because of the State of Texas Assessments of Academic Readiness (STAAR) test. That’s the priority.” Teachers of students (Grades 3–5) who participate in STAAR testing mentioned the pressure they felt to ensure that their students receive passing scores on the state’s standardized tests. They noted that, during testing time, physical activity opportunities, such as recess, had been cut back or removed to provide additional instruction time to students. In contrast, teachers of younger students (Grades K–2), who do not participate in STAAR testing, were able to provide more opportunities for physical activity. As explained by a classroom teacher:

I think that the younger grades, the teachers feel.. because they don’t have that high-stakes test, they have a little bit more ease that [they] can stay outside a little bit longer, whereas the third, fourth and fifth [grades], they have the STAAR test, and they’re concerned—‘I need to make sure I cover my content. I need to make sure that the kids are learning what they need to learn.’ So, I feel they may be giving their kids a little less [physical activity] time than others are.

Due to the increasing emphasis on testing, participants also reported little flexibility with the school day schedule. Being able to identify how physical activity opportunities would fit into the existing school day structure was a concern reported by multiple participants. An assistant principal noted:

So essentially, the number one is.. where are we going to take the time from.. to allocate time to do that, knowing already that we have all these things we have to teach, as our curriculum dictates? This is where.. we have standards and outcomes we have to have our kids be at. And, so, really considering how we can manage that time to do both, like, provide those opportunities and also meet the outcomes and the benchmarks that we have from the district, as well.

### Theme 3: Seeing the benefits of physical activity opportunities motivates school staff toward implementation

Being able to observe that physical activity opportunities were leading to desired outcomes was key to increasing implementation. Staff described how they observed the impact of physical activity on academics and classroom behavior. A PE teacher explained how classroom teachers see the benefits of using classroom-based physical activity approaches, “Teachers that use movement not only does it increase focus—behavior issues decrease because they’re having hands-on experience; they’re having fun.” In addition, some teachers who initially opposed physical activity began to advocate for opportunities after observing the benefits. An assistant principal described the reaction of one of her teachers after experiencing a motor lab during summer school:

I had a teacher from here that thought [the motor lab] was—and she had no problem telling me—she thought it was a bunch of bunk. And she taught summer school, and she came back and said, “We need a motor lab because you’re not going to believe how much better my kids were coming back from there. A lot of people think you take them in a recess, and they come back, and they’re still jumping and hyper. It’s really not the case.

Participants also shared that seeing their peers implement physical activity opportunities and the results that they achieved was an effective approach for motivating them to do the same. For example, a classroom teacher described the connection between observing benefits and the motivation for implementation:

When one teacher comes in and sees the benefits of it.. Most teachers do a 20-minute rotation, and then they go back to the classroom. But once they’re persuaded and they tell their teammates, their teammates are motivated to come and use the motor lab at least once a week.

### Theme 4: Staff buy-in is critical to the implementation process

Participants frequently spoke about the connection between staff buy-in and implementation. Participants indicated that buy-in should come from both teachers and school administrators for implementation to be successful. When asked whether mandating implementation using a top-down approach would work, a principal responded, “Well, it could. It could. But then the buy-in isn’t quite there, and the implementation won’t happen as smoothly.” Instead, buy-in was more likely to be achieved, and more quickly, when schools had a group of committed teachers leveraging the interest of other staff currently providing physical activity opportunities. According to a classroom teacher:

If you have a group of people who are committed to something that’s really good, then it will spread faster that way.. it’s better to have buy-in by having a nucleus of people who are passionate about it.

Several characteristics describe school staff who were more likely to buy in, including being passionate about physical activity in their own lives and committed to addressing students’ health needs. Further, staff who were considered more innovative and of a younger age were perceived to be more likely to buy in, whereas older teachers appeared to be less willing to change from the traditional teaching practices (i.e., sitting at a desk in the classroom). A classroom teacher stated:

This is going to be tough—this is going to be a tough buy-in just because—I don’t know, our staff is older. We have a lot of veteran teachers. I don’t even know how to word it. It’s just set in their ways, and they don’t want to do anything, any different.

Participants recalled multiple approaches for increasing staff buy-in. One simple but effective approach was to promote opportunities that were easy for teachers to use. For example, during summer school, one school repurposed an unused classroom into a motor lab to prevent the need for daily setup and teardown in the gymnasium thus saving teachers valuable time. Another effective approach was having staff experiment with multiple physical activity resources before committing to them. Experimentation helped staff determine which opportunities they felt would fit at their school, and in turn, increased their motivation to support and implement them.

We’re trying—a lot of it is example, showing them, we put that out there, and all of a sudden, we kind of had interest. And we found a lot of people that we thought would be negative towards it weren’t. So, seeing it, having somebody guinea pig and—I’m guinea pig and it’s fun—and go through the—that didn’t work, that didn’t work—the trials of it, and then saying, ‘Okay, we’ve narrowed it down these three things we think work really well.’ And having people, or us, like a group of people that are willing to do that, I think is beneficial.

## Discussion

This study explored barriers to and facilitators of the implementation of school-based physical activity opportunities, using deductive and inductive approaches. Our analysis led to the emergence of four themes, three of which are related to subcomponents (inter- and intra-organizational relationships, priority, and observability) of the R = MC^2^ heuristic and one theme (staff buy-in) that was not part of the heuristic but warrants further attention. Participants described how relationships with internal and external partners facilitated the implementation of physical activity opportunities through access to equipment, facilities, and other resources. Additionally, teachers and staff were more motivated to implement opportunities when they directly observed the benefits of physical activity. However, school staff also described barriers to implementing physical activity opportunities including a lack of time during the school day and competing school priorities such as optimizing standardized test scores. Establishing staff buy-in helped overcome many implementation challenges, especially at schools where teachers and administrators worked together to achieve this.

The R = MC^2^ heuristic posits that intra- and inter-organizational relationships have an impact on an organization’s implementation capacity [27]. Consistent with past studies, our findings also indicate the importance of good working relationships when implementing school-based physical activity opportunities [19, 44, 45]. Schools looking to improve readiness may consider establishing a district-wide collaborative to further support physical activity implementation. Collaboratives focus on the sharing of best practices and working together to develop innovative ideas to increase implementation capacity [46] and can be hosted as face-to-face meetings or web-based activities thereby fostering a spirit of inclusion and ensuring all schools have access to implementation resources/materials. In addition, working with external partners (i.e., community organizations) was critical to enhancing the quality, quantity, and diversity of physical activity opportunities offered. Resource-sharing agreements are an effective strategy that schools can utilize to develop (or sustain) relationships with community partners [47–49]. These agreements allow schools to share their facilities with local organizations (e.g., Boys & Girls Clubs) or gain access to community resources (e.g., pools at YMCA) to offer additional out-of-school time physical activity opportunities at low or no cost.

Observability, defined as the ability to see results that lead to desired outcomes, is another subcomponent featured in the R = MC^2^ heuristic that contributes to an organization’s motivation to implement an innovation [50]. The degree to which teachers were able to see physical activity opportunities leading to academic, behavioral, and physical benefits appeared to facilitate implementation. In line with previous research, we found that teachers were more receptive to providing physical activity opportunities when they could see improvements in students’ learning and academic performance [11, 51]. Encouraging teachers and school staff to share firsthand implementation experiences can promote observability and an understanding of what physical activity opportunities work best in a particular context. This information can be disseminated through teacher in-service or professional development meetings and tailored to the implementation stage (i.e., pre-implementation, adoption, active implementation, and sustainability) to increase school-wide motivation and readiness.

Based on the R = MC^2^ heuristic, the priority placed on physical activity opportunities by schools is hypothesized to influence their motivation toward implementation. The school time that was allocated for students to participate in physical activity was found to regularly compete with the time given to other educational priorities. Academic performance and instruction time were two major priorities that competed with physical activity opportunities. For example, multiple participants stated that physical activity opportunities such as recess were removed from the school day to allow time to prepare for the state’s standardized tests. Research shows that physical activity can help to improve students’ time spent on-task, which is supportive of better learning [52, 53]. For this reason, schools should emphasize students’ readiness to learn and the quality of time allocated to instruction as opposed to the amount of time only. This can be done by communicating to educators how physical activity stimulates brain development and benefits learning [54]. Schools should also explore ways to integrate physical activity into the school day to complement the academic curriculum, rather than compete with it.

Staff buy-in, which is not explicitly captured in the R = MC^2^ heuristic, is an additional factor that participants stated was essential to implementation. Our findings align with the existing buy-in literature which emphasizes the importance of buy-in to program success [55, 56] and that greater buy-in is associated with easier and more positive experiences with implementation [57]. Moreover, our findings add that staff buy-in is related to a school’s motivation toward implementing physical activity opportunities. Specifically, improving *observability* (e.g., showing teachers the results of implementing physical activity opportunities) and allowing *trialability* (e.g., having teachers test different physical activity opportunities before committing to implementation) led to greater staff buy-in and facilitated implementation. Our data support the value of targeting motivation-related subcomponents (from the R = MC^2^ heuristic) early in the implementation process to drive interest among teachers and staff, as well as highlight the need for physical activity opportunities. However, more school-based research examining the relationship between motivation, staff buy-in, and implementation is needed to determine optimal ways for achieving buy-in.

### Strengths and limitations

This study possesses several strengths. First, we used deductive and inductive approaches when coding the data to gain a comprehensive understanding of the barriers to and facilitators of implementation in the school setting and how they align with the R = MC^2^ heuristic. This approach was theoretically informed but also allowed for the identification of concepts not currently captured in the readiness framework (e.g., staff buy-in). Additionally, the interviews explored a broad range of physical activity opportunities (e.g., recess, PE, before- and after-school programs). Compared to assessing the implementation of a single opportunity in isolation, collecting information on multiple opportunities provided a holistic view of implementation in schools that aligns with real-world approaches.

Our study also has several limitations. First, it is possible that the coders’ subjectivity and awareness of the R = MC^2^ heuristic may have influenced the results of the inductive coding process. To help reduce the risk of bias, the research team met regularly throughout the coding process to review and discuss how codes were being applied. Second, in the current study, the researchers coded excerpts of interview transcripts to not replicate work already completed in the parent study. The parent study used a hierarchical coding structure, which allowed the identification of passages connected to adoption, implementation, and/or sustainability. Thus, there is a chance that relevant information from other sections of the transcripts may not have been included in the excerpts. Third, interviews were completed with a convenience sample of school staff. Although we aimed to connect with a diverse sample, it is possible that we connected with individuals more likely to speak positively about physical activity. Fourth, the parent study interviews and data collection took place prior to the COVID-19 pandemic. Although schools continue to face many of the same challenges, our findings may differ from subsequent studies given that the educational landscape has changed since the advent of COVID-19. Finally, each subcomponent from the R = MC^2^ heuristic was coded at least twice, suggesting relevancy to implementation. However, our results include descriptions of only highly relevant codes in the context of implementing school-based physical activity opportunities given the structure of the interviews and multiple topics covered.

## Conclusions

Many factors contribute to schools’ readiness to implement physical activity opportunities successfully. We found that internal and external relationships as well as observing the benefits of physical activity opportunities were essential to implementation readiness. In addition, implementation readiness was hindered by competing school priorities and a lack of staff buy-in. These insights can facilitate the selection of readiness-building strategies to improve organizational capacity and ensure high-quality implementation of physical activity opportunities in schools.

### Electronic supplementary material

Below is the link to the electronic supplementary material.

**Supplementary Material 1:** Interview Guide

## Data Availability

The datasets used and/or analyzed during the current study are available from the corresponding author upon reasonable request.

## References

[1] National Center for Education Statistics (. Fast Facts: Back-to-School Statistics. 2021; Available at: nces.ed.gov/fastfacts/display.asp?id = 372#K12-enrollment. Accessed September 8, 2021.

[2] Kohl HW, Cook HD (2013). Educating the student body: taking physical activity and physical education to school.

[3] World Health Organization. Global Action Plan on Physical Activity 2018–2030: More Active People for a Healthier World.; 2018.

[4] Seo D, King MH, Kim N, Sovinski D, Meade R, Lederer AM (2013). Predictors for moderate- and vigorous-intensity physical activity during an 18-month coordinated school health intervention. Prev Med.

[5] Sallis JF, McKenzie TL, Conway TL, Elder JP, Prochaska JJ, Brown M (2003). Environmental interventions for eating and physical activity: a randomized controlled trial in middle schools. Am J Prev Med.

[6] Pate RR, Saunders R, Dishman RK, Addy C, Dowda M, Ward DS (2007). Long-term effects of a physical activity intervention in high school girls. Am J Prev Med.

[7] Simon C, Wagner A, DiVita C, Rauscher E, Klein-Platat C, Arveiler D et al. Intervention centred on adolescents’ physical activity and sedentary behaviour (ICAPS): concept and 6-month results. Int J Obes Relat Metab Disord 2004 -11;28 Suppl 3:S96-S103. 10.1038/sj.ijo.0802812 15543228

[8] Day RE, Sahota P, Christian MS (2019). Effective implementation of primary school-based healthy lifestyle programmes: a qualitative study of views of school staff. BMC Public Health.

[9] Shoesmith A, Hall A, Wolfenden L, Shelton RC, Powell BJ, Brown H (2021). Barriers and facilitators influencing the sustainment of health behaviour interventions in schools and childcare services: a systematic review. Implement Sci.

[10] Gorely T, Harrington DM, Bodicoat DH, Davies MJ, Khunti K, Sherar LB (2019). Process evaluation of the school-based girls active programme. BMC Public Health.

[11] van den Berg V, Salimi R, de Groot RHM, Jolles J, Chinapaw MJM, Singh AS. "It's a Battle? You Want to Do It, but How Will You Get It Done?": Teachers' and principals' perceptions of implementing additional physical activity in school for academic performance. Int J Environ Res Public Health 2017 Sep 30;14(10):1160. 10.3390/ijerph14101160. 10.3390/ijerph14101160 PMC5664661 28973967

[12] Szeszulski J, Walker T, Robertson M, Cuccaro P, Fernandez ME (2020). School Staff’s perspectives on the adoption of Elementary-School Physical Activity approaches: a qualitative study. Am J Health Educ.

[13] Campbell EJ, Lee Olstad D, Spence JC, Storey KE, Nykiforuk CIJ (2020). Policy-influencer perspectives on the development, adoption, and implementation of provincial school-based daily physical activity policies across Canada: a national case study. SSM Popul Health.

[14] Economos CD, Mueller MP, Schultz N, Gervis J, Miller GF, Pate RR (2018). Investigating best practices of district-wide physical activity programmatic efforts in US schools- a mixed-methods approach. BMC Public Health.

[15] Bogart LM, Fu CM, Eyraud J, Cowgill BO, Hawes-Dawson J, Uyeda K (2018). Evaluation of the dissemination of SNaX, a middle school-based obesity prevention intervention, within a large US school district. Transl Behav Med.

[16] Hastmann TJ, Bopp M, Fallon EA, Rosenkranz RR, Dzewaltowski DA (2013). Factors influencing the implementation of organized physical activity and fruit and vegetable snacks in the HOP’N after-school obesity prevention program. J Nutr Educ Behav.

[17] Hayes CB, O’Shea MP, Foley-Nolan C, McCarthy M, Harrington JM (2019). Barriers and facilitators to adoption, implementation and sustainment of obesity prevention interventions in schoolchildren- a DEDIPAC case study. BMC Public Health.

[18] Egan CA, Webster CA, Stewart GL, Weaver RG, Russ LB, Brian A et al. Case study of a health optimizing physical education-based comprehensive school physical activity program. Evaluation and Program Planning 2019 February 1,;72:106–17. 10.1016/j.evalprogplan.2018.10.006 30326329

[19] Dyrstad SM, Kvalø SE, Alstveit M, Skage I (2018). Physically active academic lessons: acceptance, barriers and facilitators for implementation. BMC Public Health.

[20] Weatherson KA, McKay R, Gainforth HL, Jung ME (2017). Barriers and facilitators to the implementation of a school-based physical activity policy in Canada: application of the theoretical domains framework. BMC Public Health.

[21] Gamble A, Chatfield SL, Cormack ML, Hallam JS (2017). Not enough time in the day: a qualitative assessment of in-school physical activity policy as viewed by administrators, teachers, and students. J Sch Health.

[22] Agron P, Berends V, Ellis K, Gonzalez M (2010). School wellness policies: perceptions, barriers, and needs among school leaders and wellness advocates. J Sch Health.

[23] Bolton KA, Kremer P, Gibbs L, Swinburn B, Waters E, de Silva A (2015). Expanding a successful community-based obesity prevention approach into new communities: challenges and achievements. Obes Res Clin Pract.

[24] Chalkley AE, Routen AC, Harris JP, Cale LA, Gorely T, Sherar LB (2018). A retrospective qualitative evaluation of barriers and facilitators to the implementation of a school-based running programme. BMC Public Health.

[25] Walker TJ, Craig DW, Pavlovic A, Thiele S, Natale B, Szeszulski J (2021). Physical activity and Healthy Eating Programming in Schools to Support Student’s Health-Related Fitness: an observational study. Int J Environ Res Public Health.

[26] Weiner BJ. A theory of organizational readiness for change. Imp Sci 2009-10-19;4:67. 10.1186/1748-5908-4-67 PMC2770024 19840381

[27] Scaccia JP, Cook BS, Lamont A, Wandersman A, Castellow J, Katz J (2015). A practical implementation science heuristic for organizational readiness: R = MC2. J Community Psychol.

[28] Damschroder LJ, Aron DC, Keith RE, Kirsh SR, Alexander JA, Lowery JC (2009). Fostering implementation of health services research findings into practice: a consolidated framework for advancing implementation science. Implement Sci.

[29] Rycroft-Malone J, Kitson A, Harvey G, McCormack B, Seers K, Titchen A (2002). Ingredients for change: revisiting a conceptual framework. Qual Saf Health Care.

[30] Kitson AL, Rycroft-Malone J, Harvey G, McCormack B, Seers K, Titchen A. Evaluating the successful implementation of evidence into practice using the PARiHS framework: theoretical and practical challenges. Implement Sci 2008-01-07;3:1. 10.1186/1748-5908-3-1 PMC2235887 18179688

[31] Nilsen P. Making sense of implementation theories, models, and frameworks. Implementation Science 3.0: Springer; 2020. p. 53–79. 10.1186/s13012-015-0242-0 PMC4406164 25895742

[32] Dias EM, Walker TJ, Craig DW, Gibson R, Szeszulski J, Brandt HM (2023). Examining readiness for implementing practice changes in federally qualified health centers: a rapid qualitative study. J Community Psychol.

[33] Splett JW, Perales K, Miller E, Hartley SN, Wandersman A, Halliday CA (2022). Using readiness to understand implementation challenges in school mental health research. J Community Psychol.

[34] Calvert HG, Lane HG, McQuilkin M, Wenner JA, Turner L. Elementary Schools’ Response to Student Wellness Needs during the COVID-19 Shutdown: A Qualitative Exploration Using the R = MC2 Readiness Heuristic. Int J Environ Res Public Health. 2021-12-27;19(1):279. 10.3390/ijerph19010279 PMC8750629 35010539

[35] Texas Education Agency. Texas Academic Performance Report 2019-20 performance reporting Division. Texas Education Agency (TEA); 2020.

[36] Walker TJ, Szeszulski J, Robertson MC, Cuccaro PM, Fernandez ME. Understanding implementation strategies to support classroom-based physical activity approaches in elementary schools: a qualitative study. Eval Program Plann 2022 -06;92:102051. 10.1016/j.evalprogplan.2022.102051 PMC9177707 35240403

[37] Walker TJ, Pfledderer CD, Craig DW, Robertson MC, Heredia NI, Bartholomew JB. Elementary school staff perspectives on the implementation of physical activity approaches in practice: an exploratory sequential mixed methods study. Front Public Health (in press) 2023. 10.3389/fpubh.2023.1193442 PMC10483115 37693726

[38] Walker TJ, Craig DW, Pfledderer CD, Robertson MC, Cuccaro P, Fumero K (2023). Observed and perceived benefits of providing physical activity opportunities in elementary schools: a qualitative study. Front Sports Act Living.

[39] Palinkas LA, Horwitz SM, Green CA, Wisdom JP, Duan N, Hoagwood K (2015). Purposeful sampling for qualitative data collection and analysis in mixed method implementation research. Adm Policy Mental Health Mental Health Serv Res.

[40] Ritchie J, Spencer L, Bryman A, Burgess RG (1994). Qualitative data analysis for applied policy research.

[41] Gale NK, Heath G, Cameron E, Rashid S, Redwood S. Using the framework method for the analysis of qualitative data in multi-disciplinary health research. BMC Med Res Methodol 2013-09-18;13:117. 10.1186/1471-2288-13-117 PMC3848812 24047204

[42] Rabin BA, Brownson RC, Haire-Joshu D, Kreuter MW, Weaver NL. A glossary for dissemination and implementation research in health. J Public Health Manag Pract 2008 Mar-Apr;14(2):117–23. 10.1097/01.PHH.0000311888.06252.bb 18287916

[43] ATLAS.ti Scientific Software Development GmbH [ATLAS.ti 22 Windows]. (2022). Retrieved from https://atlasti.com.

[44] Greaney ML, Hardwick CK, Spadano-Gasbarro JL, Mezgebu S, Horan CM, Schlotterbeck S et al. Implementing a multicomponent school-based obesity prevention intervention: a qualitative study. J Nutr Educ Behav 2014 Nov-Dec;46(6):576–82. 10.1016/j.jneb.2014.04.293 24878150

[45] van Nassau F, Singh AS, Broekhuizen D, van Mechelen W, Brug J, Chinapaw MJM (2016). Barriers and facilitators to the nationwide dissemination of the Dutch school-based obesity prevention programme DOiT. Eur J Public Health.

[46] Nadeem E, Olin SS, Hill LC, Hoagwood KE, Horwitz SM. Understanding the components of quality improvement collaboratives: a systematic literature review. Milbank Q 2013 -06;91(2):354–94. 10.1111/milq.12016 PMC3696201 23758514

[47] Rabin BA, Glasgow RE, Kerner JF, Klump MP, Brownson RC (2010). Dissemination and implementation research on community-based cancer prevention: a systematic review. Am J Prev Med.

[48] Rugs D, Hills HA, Moore KA, Peters RH. A community planning process for the implementation of evidence-based practice. Eval Program Plann 2011 -02;34(1):29–36. 10.1016/j.evalprogplan.2010.06.002 20674026

[49] Reed RG, Fong SY, Pearson TA. Role of a central laboratory in implementing national cholesterol education panel guidelines in rural practices: model system for managed care. Clin Chem 1995 -02;41(2):271–4. 7874781

[50] Rogers EM (2003). Diffusion of innovations.

[51] McMullen JM, Martin R, Jones J, Murtagh EM (2016). Moving to learn Ireland– Classroom teachers’ experiences of movement integration. Teacher and Teaching Education.

[52] Watson A, Timperio A, Brown H, Hesketh KD (2019). Process evaluation of a classroom active break (ACTI-BREAK) program for improving academic-related and physical activity outcomes for students in years 3 and 4. BMC Public Health.

[53] Bartholomew JB, Jowers EM, Roberts G, Fall A, Errisuriz VL, Vaughn S (2018). Active learning increases children’s physical activity across demographic subgroups. Translational J Am Coll Sports Med.

[54] Hillman CH, Pontifex MB, Raine LB, Castelli DM, Hall EE, Kramer AF. The effect of acute treadmill walking on cognitive control and academic achievement in preadolescent children. Neuroscience 2009-3-31;159(3):1044–54. 10.1016/j.neuroscience.2009.01.057 PMC2667807 19356688

[55] Larsen T, Samdal O. Facilitating the implementation and sustainability of second step. Scandinavian J Educational Res 2008-04-01;52(2):187–204.

[56] Storey KE, Montemurro G, Flynn J, Schwartz M, Wright E, Osler J et al. Essential conditions for the implementation of comprehensive school health to achieve changes in school culture and improvements in health behaviours of students. BMC Public Health 2016-11-02;16(1):1133. 10.1186/s12889-016-3787-1 PMC5094006 27806692

[57] Olarte DA, Stock M, Sutton M, Scott M, Koch PA, Gustus S (2022). Teachers’ experiences implementing a School Wellness Initiative in Anchorage, AK: a qualitative study. J Acad Nutr Diet.

