# Supplementary material for: Using the R = MC2 heuristic to understand barriers to and facilitators of implementing school-based physical activity opportunities: a qualitative study
Source: BMC Public Health. 2024 Jan 17;24:207. doi: 10.1186/s12889-024-17744-2 (PMC10792959; doi:10.1186/s12889-024-17744-2)
Supplement: Supplementary file 1 — Supplementary Material 1: Interview Guide [file 12889_2024_17744_MOESM1_ESM.docx]

**In-Depth Individual Interview Guide**

Warm-up Questions

1. What is your current position?
2. How long have you been working at your school?
3. What do you like most about your position?

Programming-related questions

1. How do you feel about the amount of physical activity students are getting at your school?
2. Tell me about the current physical activity policies at your school
   1. Probe: how well are policies adhered to?
3. Tell me about the current physical activity approaches at your school
   1. Probe: How effective are the approaches

Implementation

1. How are physical activity approaches selected to be used?
   1. Probe: who is involved in the decision-making?
   2. Probe: what resources are available to support decision-making?
2. How do you feel about the process of delivering programs?
   1. Probe: who is involved in delivering programs
   2. Probe: what resources are available?
   3. Probe: what strategies help delivery?
3. What makes it difficult to deliver physical activity programs?
4. What makes it easier to deliver physical activity programs?
5. What makes an approach stick or be sustained?
